# Multiparametric physicochemical analysis of a type 1 collagen 3D cell culture model using light and electron microscopy and mass spectrometry imaging

**DOI:** 10.1038/s41598-025-93700-3

**Published:** 2025-03-20

**Authors:** Camilla Dondi, Dimitrios Tsikritsis, Jean-Luc Vorng, Gina Greenidge, Ibolya E. Kepiro, Natalie A. Belsey, Greg McMahon, Ian S. Gilmore, Maxim G. Ryadnov, Michael Shaw

**Affiliations:** 1 National Physical Laboratory, Hampton Road, Teddington, TW11 0LW UK; 2 School of Chemistry and Chemical Engineering, University of Surrey, Guildford, GU2 7XH UK; 3 UCL Hawkes Institute and Department of Computer Science, University College London, London, UK

**Keywords:** Optical imaging, Cellular imaging, Cytoskeleton, Imaging, Mass spectrometry, Optical spectroscopy, Microscopy

## Abstract

Three-dimensional cell culture systems underpin cell-based technologies ranging from tissue scaffolds for regenerative medicine to tumor models and organoids for drug screening. However, to realise the full potential of these technologies requires analytical methods able to capture the diverse information needed to characterize constituent cells, scaffold components and the extracellular milieu. Here we describe a multimodal imaging workflow which combines fluorescence, vibrational and second harmonic generation microscopy with secondary ion mass spectrometry imaging and transmission electron microscopy to analyse the morphological, chemical and ultrastructural properties of cell-seeded scaffolds. Using cell nuclei as landmarks we register fluorescence with label-free optical microscopy images and high mass resolution with high spatial resolution secondary ion mass spectrometry images, with an accuracy comparable to the intrinsic spatial resolution of the techniques. We apply these methods to investigate relationships between cell distribution, cytoskeletal morphology, scaffold fiber organisation and biomolecular composition in type I collagen scaffolds seeded with human dermal fibroblasts.

**Supplementary Information:**

The online version contains supplementary material available at 10.1038/s41598-025-93700-3.

## Introduction

Analysis of in vitro eukaryotic cell cultures forms the basis of much of our empirical understanding of cell and tissue physiology, pathologies and cellular responses to therapeutic and toxic agents. Two-dimensional (2D) monolayer cultures, in which cells are grown on a (typically glass or plastic) planar substrate are simple and widely accessible, but do not represent many features of the in vivo context. Three-dimensional (3D) culture models can better replicate the native growth environment and provide a more accurate representation of physiology^1^, allowing cells to interact and behave as they would in vivo^2^. 3D models can bridge gaps between traditional in vitro studies and clinical applications and enable more reliable preclinical testing of therapeutic efficacy and toxicity^3^, facilitating the development of new, personalised treatments^4–8^. Engineering functional tissues and organs in the laboratory offers new opportunities to treat diseases, repair or replace tissues, address organ shortages and enhance patient care.

3D cell cultures are complex, heterogenous systems constituted by dynamic cell-cell and cell-matrix interactions. The cellular microenvironment plays a crucial role in regulating cell fate^9^ and replicating the complexity of the in vivo extracellular matrix (ECM) in the laboratory enables study and control of cues that can, alone or in unison, drive cellular behaviours. A variety of hydrogels have been explored as matrices to support 3D cell cultures^10,11^. Biocompatible hydrogels, capable of encapsulating cells and supporting cell growth, have been derived from decellularized tissues and proteins, synthetic polymers, plant-derived alginates, and synthetic peptides. Collagen is the most abundant mammalian protein. It is evolutionally conserved and universally forms the basis of the native ECM in connective tissues; in which supramolecular collagen assemblies regulate cell behaviour and influence structural and mechanical properties^12,13^. Cell-seeded collagen scaffolds are widely used as models for tissue damage and remodelling to investigate the biochemical and biomechanical exchanges that occur between collagen and the different cells involved in the wound healing process, with fibroblasts the principal cell type responsible for synthesising the new ECM^14^.

A variety of culture systems and analytical methods have been applied to investigate how the chemical, mechanical and microstructural properties of 3D scaffolds and hydrogels influence fibroblast behaviour^15,16^. High-resolution imaging techniques are widely employed to investigate spatial heterogeneity in 3D cell culture models^17^. Its capacity for minimally invasive, volumetric, live cell imaging makes light microscopy particularly well suited to capturing the structural and dynamic properties of these systems. Collagen remodelling and deposition by fibroblasts in culture models has been visualised using multiphoton imaging, phase contrast and fluorescence microscopy^18–20^. Confocal laser scanning fluorescence microscopy (CLSFM) has been applied to visualise fluorescently tagged collagen fibres and migrating cells^21^. Newer fluorescence microscopy methods such as light sheet^22^ and super-resolution approaches^23^ enable imaging of the dynamic behaviour of cells in collagen scaffolds^24^ with improved spatio-temporal resolution over larger scales of space and time. Fibrillar collagen has a large nonlinear susceptibility and (label-free) second harmonic generation (SHG) microscopy can be used to visualise the structure and arrangement of collagen fibres and fibrils^25^ and investigate scaffold remodeling^26^. Spontaneous and coherent Raman microscopy techniques can detect specific chemical bonds and analyse the chemical composition of cells and the extracellular matrix^27^. Mass spectrometry imaging^28^ can detect and localise molecular fragments associated with biomaterials, cellular components, and metabolites. Scanning electron microscopy (SEM) is widely used for characterising scaffold microstructure and cell attachment^16^, whilst micro-computed tomography (µCT) can capture 3D images of the scaffold structure from which properties including pore size can be determined^29^. Atomic force microscopy (AFM) is able to capture high-resolution structural^30^ and mechanical information from individual collagen fibrils^31^. Whilst limited to the analysis of thin sections, transmission electron microscopy (TEM) can be applied to measure the size and distribution of individual collagen fibrils^32^.

**Fig. 1:**
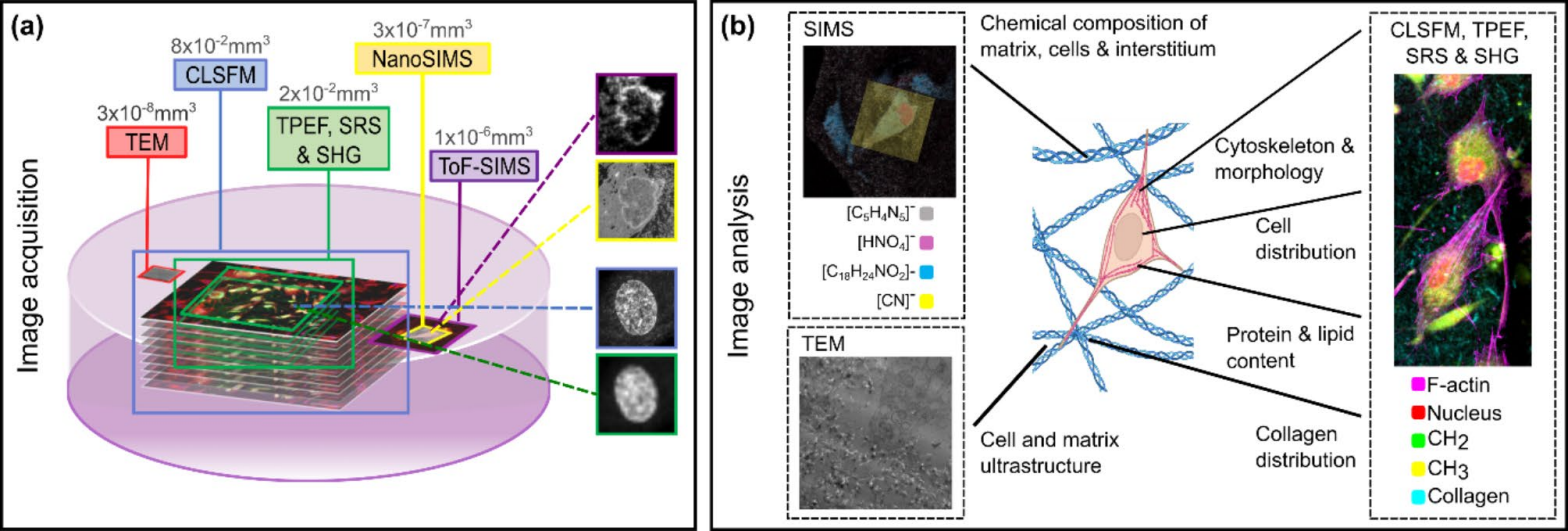
Acquisition and analysis of multiparametric image data for characterization of cell-seeded scaffolds. (**a**) Illustration of the volume sampled using the different techniques included in the multimodal imaging workflow (typical image volumes shown above the corresponding method label). After minimal chemical fixation, correlative CLSFM, TPEF, SRS and SHG images are captured over a common 3D ROI. Following resin-embedding, correlative NanoSIMS and ToF-SIMS analysis is performed on the same ROI from thick (250 nm) sections. Thinner (70 nm) sections cut from the same block are analysed using TEM. Inset images show (from top) ToF-SIMS, NanoSIMS, CLSFM and TPEF images of cell nuclei used to generate landmarks for image registration. (**b**) The features of the cell-seeded scaffold system accessible using the different imaging techniques.

A single imaging technique is limited in spatio-temporal resolution and the type and quantity of information it can provide. The intrinsic variability and heterogeneity of biological systems makes it challenging to combine and interpret data captured from different samples prepared to meet the requirements of different imaging techniques. Multimodal and correlative imaging approaches seek to apply different imaging modalities for multiparametric analysis^33^ of a common sample. As well as removing the problem of inter-sample variability, imaging the same region of interest (ROI) within a common sample using different methods also permits qualitative and quantitative analysis of signal co-localisation^34^. 3D cell cultures present particular challenges for multimodal imaging due to their size, heterogeneity and complexity. Correlative light and electron microscopy techniques have been applied for investigating organoids^35^ and 3D culture systems including collagen fibres and fibroblasts^36^. The additional use of x-ray µCT has been used to visualise opaque, porous scaffold systems^37^. However, these workflows only capture a subset of the information required to characterise 3D culture systems and elucidate relationships between cells and the ECM. To address these limitations we developed a multimodal, partially-correlative high-resolution imaging workflow (Fig. 1a) which we applied to investigate the structural, morphological, and chemical properties of human dermal fibroblasts (HDFs) seeded in type I collagen scaffold (Fig. 1b). We used CLSFM, two-photon excitation fluorescence (TPEF), stimulated Raman scattering (SRS) and SHG microscopy to visualise the distribution of cells, cytoskeletal organisation, the relative abundance of lipids and proteins within different cellular compartments and the local ECM structure in minimally fixed scaffolds. Following resin embedding, curing and staining, thin sections cut from the same samples were analysed using sequential Time of Flight (ToF) and Nanoscale (Nano) secondary ion mass spectrometry (SIMS) imaging to identify molecular fragments associated with the scaffold and cellular organelles. Further sections were analysed using transmission electron microscopy (TEM) to visualise the encapsulation of HDFs in the collagen scaffold. Fluorescent tags and molecules associated with cell nuclei were used to generate landmarks for registration of CLSFM with multiphoton images and ToF-SIMS with NanoSIMS datasets, with a measured mean target registration error (TRE) comparable to, or less than, the nominal lateral spatial resolution of the individual images. In this article we describe the experimental and computational methods used within this multimodal imaging workflow and discuss their merits and limitations. We analyse image data to elucidate different features of a 3D collagen-HDF culture system and discuss the potential for further development and application of extended multimodal and correlative imaging workflows for wider analysis of 3D cell cultures.

## Results

### Accuracy of fiducial-free registration of multimodal optical and multimodal SIMS image data using nuclear features

**Fig. 2:**
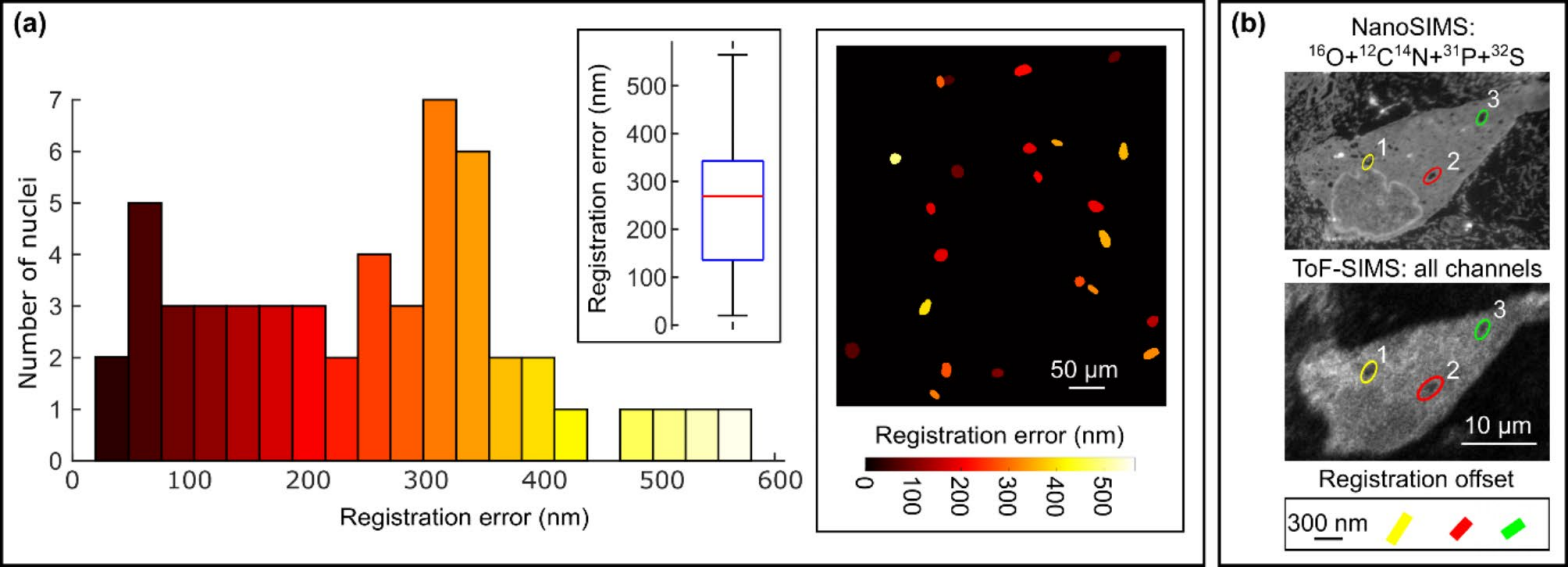
Measured target registration errors for fluorescence microscopy and SIMS image data. (**a**) Histogram of lateral target registration errors between CLSFM and TPEF images, based on centre of mass locations of 53 nuclei from three different image datasets (left panel). Inset plot shows 25th and 75th percentiles (136.2 nm and 342.9 nm, blue box), median (269 nm, red line) and minimum and maximum measured registration errors (20.0 nm and 564.5 nm, solid black lines). Segmented nuclei from a single image dataset are shown coloured by measured lateral registration error (right panel). (**b**) Registered NanoSIMS (upper panel) and ToF SIMS (lower panel) images of the same HDF cell. The lateral target registration error measured using the features highlighted in the elliptical ROIs is 289 nm ± 62 nm.

Four type I collagen scaffolds samples seeded with HDFs were prepared on gridded coverslips to facilitate identification of common ROIs using different optical microscope systems. Maximum intensity (z) projections computed from CLSFM, TPEF, SRS and SHG focal series (z-stacks) were registered to allow visualization of multimodal optical images of the same ROI. To quantify the registration accuracy we defined a target registration error (TRE) measure based on the Euclidean distance between the centre of mass (CoM) of corresponding nuclei in CLSFM and TPEF image data. Cell nuclei in registered image pairs were segmented using a pretrained object detection model implemented in the ImageJ plugin StarDist^38^ (version 0.3.0). The location of the CoM of each detected nucleus was computed within the ROI returned by the nuclear segmentation model. CoM coordinates were imported into MATLAB (R2023b, Mathworks) with corresponding nuclei in CLSFM and TPEF images identified by minimization of the Euclidean distance between their CoM locations. For 53 cell nuclei across three different HDF-collagen samples we found a mean TRE of 250 nm, slightly larger than the lateral spatial resolution and pixel size of the CLSFM images (nominally 200 nm and 180 nm respectively) and significantly less than the lateral spatial resolution and pixel size of the TPEF images (nominally 400 nm and 910 nm respectively). This TRE measure includes contributions from the nuclear CoM estimation and the registration method used to correct geometric differences between the CLSFM and TPEF images caused by differences in sample mounting, sample instability and the imaging systems themselves. TRE values for individual nuclei ranged from 20 nm to 564 nm and errors varied significantly across each image field due to the non-uniform distribution of registration landmarks and the complex deformation of the image space coordinates between the two different microscope systems (Fig. 2a).

A similar method was used to estimate the TRE for the registered ToF-SIMS and NanoSIMS images of an HDF encapsulated by the scaffold (Fig. 2b). Total ion images were computed by summing the^16^O, ^12^C^14^N, ^31^P and ^32^S channels of the NanoSIMS data set and all 753 channels of the ToF-SIMS data set. Several large, dark (low signal) voids are visible within the cell body in both NanoSIMS and ToF-SIMS images (highlighted by red, yellow and green ellipses). A TRE measure was derived from the Euclidean distance between the CoM of corresponding voids (computed within an elliptical ROI defined on an inverted version of each image). The distance offsets between void CoMs in NanoSIMS and ToF SIMS images were 358 nm, 250 nm, 252 nm, corresponding to a mean TRE of 289 nm. This is approximately twice the size of the ToF-SIMS image pixels and, as with optical microscopy images, includes contributions from the object centroid estimation as well errors from the registration process. However, the lower image contrast and signal-to-noise ratio, particularly for the ToF-SIMS image data, means the object centroiding error likely makes a larger contribution to the estimate than for the optical image data.

### Correlative fluorescence, SRS and SHG microscopy reveals relationships between scaffold density, cell distribution, morphology and protein/lipid composition

**Fig. 3:**
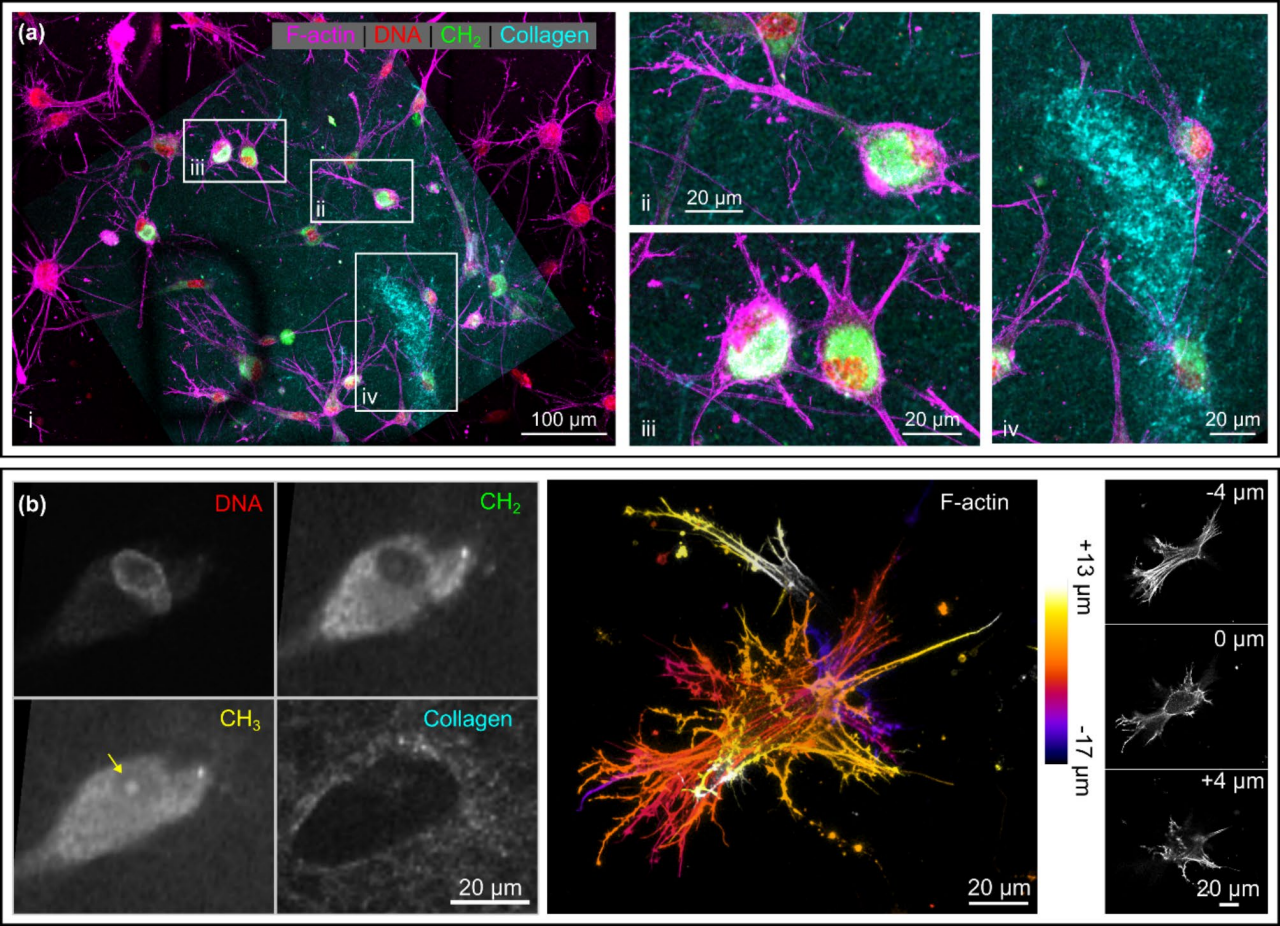
Correlative optical microscopy images of HDFs in collagen scaffolds. (**a**) Maximum intensity (z) projection of registered CLSFM, TPEF, SRS and SHG image data showing HDF cells in a collagen scaffold. The magnified views in panels (**ii**) and (**iii**) show how combining CLSFM images of F-actin (magenta) with SRS CH_2_ images (green) enable simultaneous visualization of the actin cytoskeleton and the lipid-rich cytoplasm. Panel (**iv**) highlights cells excluded from high density regions of the scaffold. (**b**) (left) Multiphoton images reveal the composition and structure of a single HDF and the niche it occupies within the scaffold. A nucleolus visible in the CH_3_ image is highlighted by the yellow arrow. (right) Colour depth projection and individual image slices from the CLSFM z-stack showing the cytoskeleton of the same cell.

**Fig. 4:**
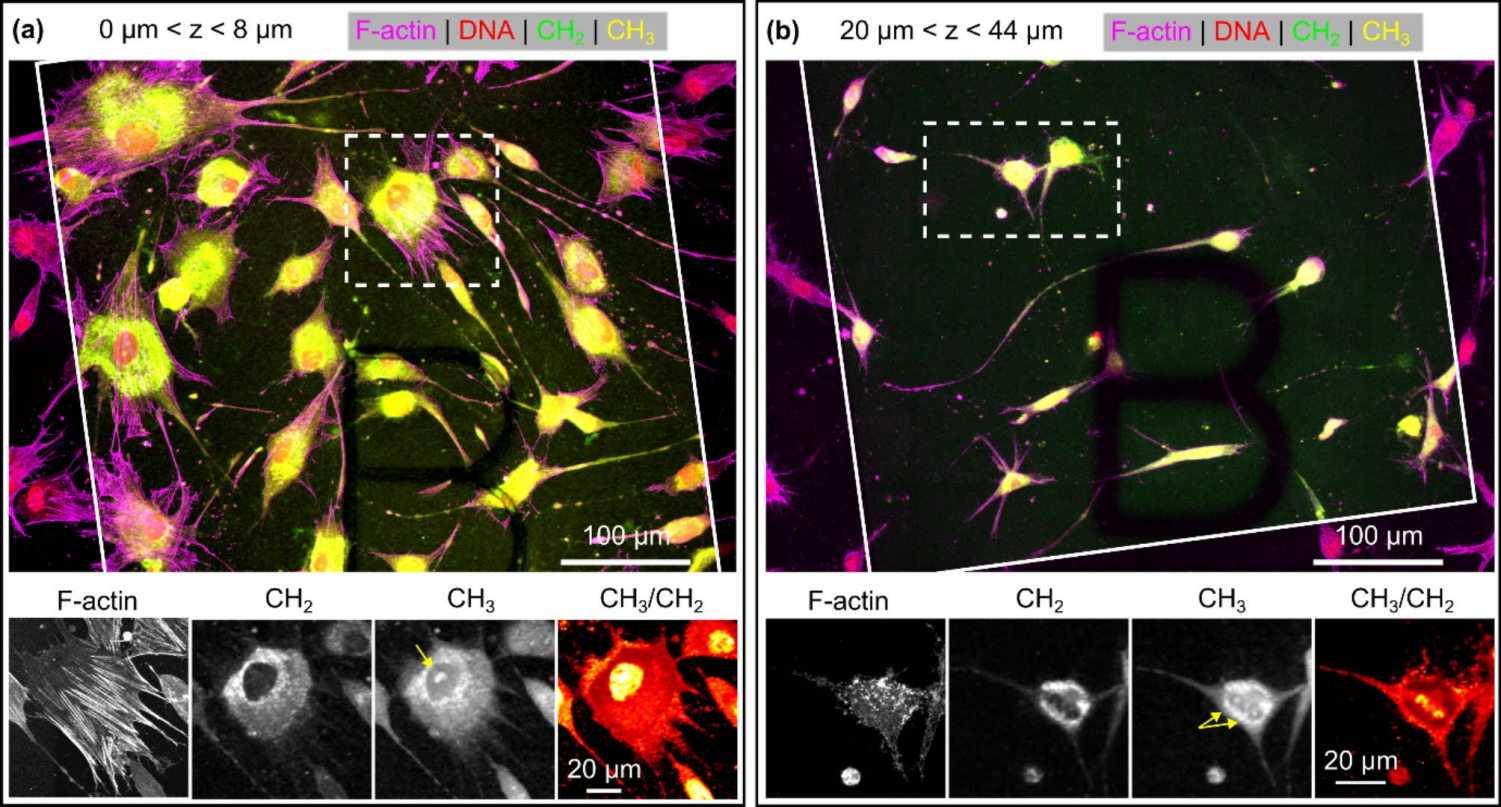
SRS and CLSM images of HDFs at different depths within a type I collagen scaffold. Lower row shows individual image channels and CH_3_/CH_2_ ratio images for single cells within boxed regions indicated above. Yellow arrows indicate nucleoli visible CH_3_ images. (**a**) Maximum intensity projection over 0–8 μm from the coverslip-gel interface. Cells adhered to the coverslip have a thin, laterally spread morphology with long actin stress fibers. A large CH_3_ (protein) rich cluster is visible inside the nucleus of the highlighted cell. (**b**) In contrast, HDFs 20–44 μm deep inside the scaffold have a multipolar (typically bipolar or tripolar) morphology with a compact elliptical cell body and a small number of prominent projections. CH_3_ rich clusters are visible inside the nucleus of the highlighted cell and the actin cortex presents as a series of puncta. Images were normalized and processed using a median filter with a radius of one pixel.

Each HDF-seeded type I collagen scaffold was visually inspected under low excitation laser power before z-stacks of two representative volumes were captured using CLSF, SRS, TPEF and SHG microscopy. Maximum intensity (z) projections from the different image modalities were spatially registered and examined to investigate different features of the 3D culture system. We observed qualitatively similar trends across all samples and include representative results in Figs. 3 and 4. Figure 3a shows images spanning a depth range from 12 μm to 48 μm above the top surface of the coverslip. HDFs display a multipolar morphology characterized by a compact, rounded cell body and long, dendritic projections. Figure 3b shows magnified images of a single cell; methyl groups within cell body give rise to an intense SRS signal corresponding to the asymmetric CH_3_ stretch (yellow). The high SHG signal (cyan) in the lower right region of the image (highlighted in subpanel iv) indicates that this region of the scaffold contains a particularly dense network of collagen fibres, which appear to exclude cell bodies and the dendritic projections from neighbouring cells. To estimate the variation in collagen density across the image field we measured the SHG signal intensity within three (12 × 12 pixel) ROIs inside this dense collagen patch and three (12 × 12 pixel) ROIs in adjacent (background) scaffold regions. Outside the collagen rich region the mean SHG signal was only 39% (32 +/- 1) of the value measured close to the centre of the patch (82 +/- 6).

Figure 3b shows magnified images of a single HDF deep inside the scaffold. The collagen-free niche occupied by the cell body is visible in the SHG image. In contrast to the compact morphology suggested by the SRS, CH_2_ and CH_3_ image channels, the high-resolution CLSFM image reveals that the cell has a stellate morphology with projections extending into the scaffold. The abundant F-actin stress fibres suggest the cell is strongly attached to the scaffold^39^. The activated phenotype^40^ indicated by this cytoskeletal morphology is also associated with upregulation of genes associated with collagen biosynthesis^41,42^. Hoechst (selective DNA stain) contains both CH_2_ and CH_3_ groups, thus it contributes to the SRS signal from endogenous CH_2_ and CH_3_ containing molecules within the cell nucleus. However, fluorescent emission visible in the TPEF image is primarily confined to the nuclear periphery, suggesting the bright punctate feature close to the centre of the nucleus is a nucleolus, visible in the CH_3_ channel owing to its relatively high protein content.

To further investigate the effect of the local ECM environment on cell morphology we examined HDFs at different locations within the same scaffold. Consistent with other studies^43^, we observed that cells close to the coverslip-scaffold interface (Fig. 4a) had a spread 2D morphology and a cytoskeleton rich in organised actin stress fibres. By contrast, cells deep inside the scaffold (Fig. 4(b)) had a compact body with a small number of long actin-rich protrusions. Both of these morphologies are indicative of the lamellipodial migration mode typical of cells in 3D and 2D^43^. To visualize the relative abundance of CH_3_ and CH_2_ groups, we derived a CH_3_/CH_2_ ratio image by dividing the normalized, background corrected, CH_3_ image by the normalized CH_2_ image. CH_2_ and CH_3_ groups are found in many different classes of biomolecules, however lipids generally contain a greater number of CH_2_ groups than CH_3_ groups and the inverse is typically true for proteins. Thus the CH_3_/CH_2_ ratio images in Fig. 4 suggest a relatively high protein concentration within the cell nucleus. The protein/lipid ratio, and the relative abundance of other groups which are detectable using Raman spectroscopy, have been shown to be sensitive to changes in biochemical components in cells and nuclei during proliferation^44^. The number, composition and distribution of nucleoli are indicative of the stage of the cell cycle^45^ and the mechanical cues provided to cells by the ECM^46^. Combining SRS and FM image data also allows visualization of binucleation in individual cells. The highlighted cell in Fig. 4b contains two prominent nucleoli, clearly visible in the CH_3_/CH_2_ ratio image, with septa visible in the corresponding CLSFM nucleus image (see Fig. S1 in supplementary material).

### Correlative SIMS analysis shows the intra and extracellular distribution of biomolecules localized to cells, organelles and the ECM.

**Fig. 5:**
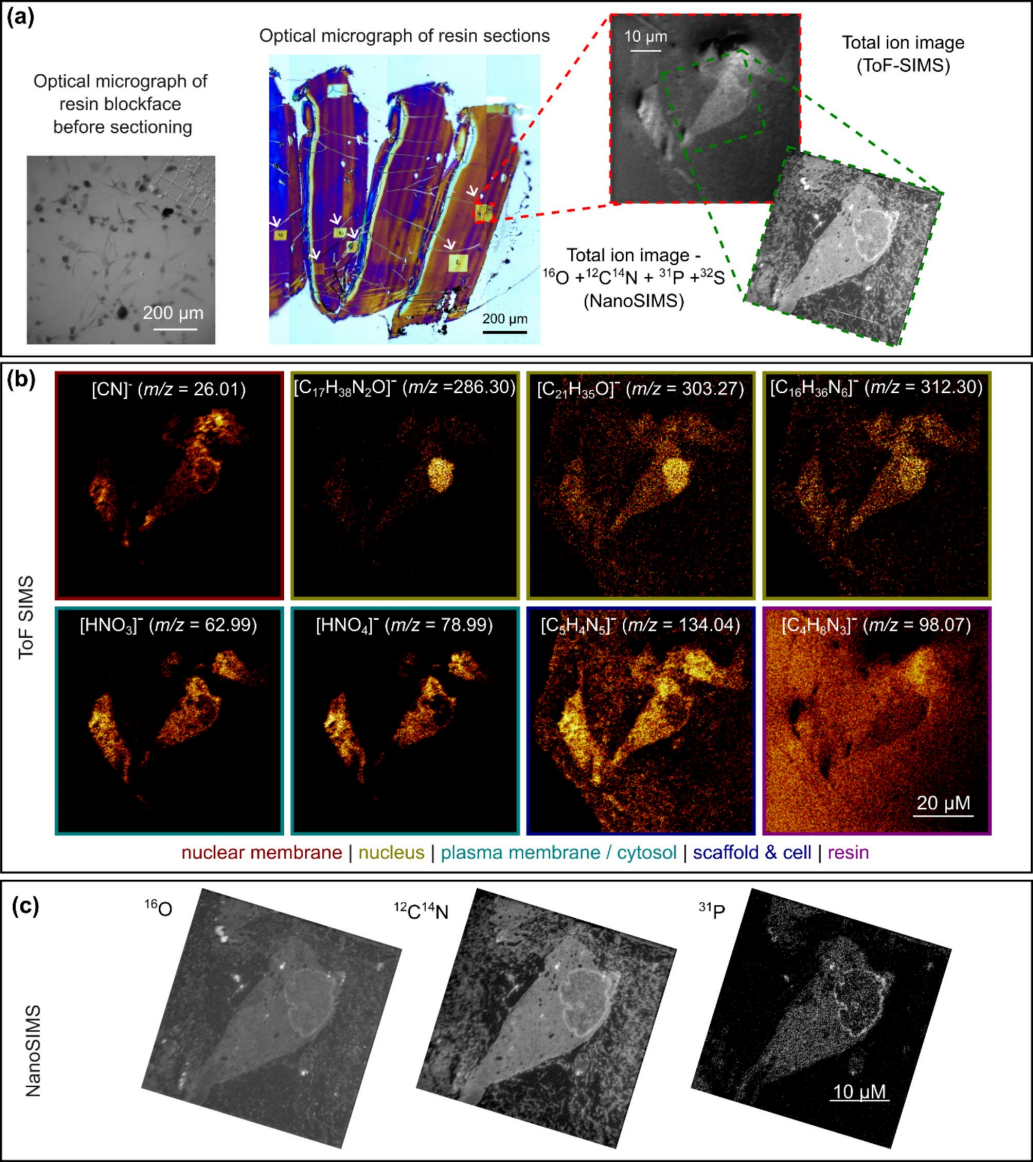
SIMS images of sections prepared from the same HDF-seeded collagen scaffold after resin embedding. (**a**) Optical image of the face of the resin block (left), brightfield images of 250 nm thick sections on a silicon wafer (white arrows indicate ToF-SIMS sputter craters) (centre) and ToF-SIMS and NanoSIMS total ion images (right). (**b**) ToF-SIMS images of molecular fragments associated with the nuclear membrane, the nucleus, the plasma membrane and/or cytosol, the scaffold and/or interstitium and the resin. (**c**) NanoSIMS images of oxygen, cyanide and phosphorus ions in the same cell. All sections were prepared from the same sample for which optical microscopy images are shown in Fig. 3a.

After acquisition of optical microscopy image data, cell-seeded collagen scaffolds were further stained, resin embedded and sectioned for SIMS and TEM analysis. Cells displaying a variety of the morphologies seen in the CLSFM and SRS image data (Figs. 3 and 4) are visible in the reflected light image of the resin block (Fig. 5a). Brightfield optical microscopy was used to select suitable ROIs within 250 nm thick sections prepared from the block for SIMS analysis. SIMS is a partially destructive technique and the square ion beam sputter craters visible following ToF-SIMS data acquisition were used to identify the same sample ROI for NanoSIMS analysis. By experimentally optimising the ToF-SIMS data acquisition it was possible to obtain a sufficient ion yield to reconstruct individual (mass-to-charge) image channels whilst retaining enough material within the ROI for subsequent NanoSIMS analysis, and stained cells are clearly visible inside the ToF-SIMS sputter craters in Fig. 5a. ToF-SIMS data were analysed to identify molecular fragments localised to different subcellular components, the ECM, and the resin. Figure 5b shows eight image channels corresponding to prominent peaks in the mass spectrum (see Fig. S2 and table S1 in supplementary material). Cyanide ions ([CN]^-^, *m/z* = 26.01)) are present throughout the cell body and at particularly high concentration close to the nuclear membrane, as confirmed in the NanoSIMS image of the same cell (Fig. 5c). Cyanide groups are associated with many different species, including fragments of amino acids, and as such [CN]^−^ ions cannot be assigned to a specific molecule. [C_17_H_38_N_2_O]^−^ (*m/z* = 286.30) and, to a lesser extent, [C_21_H_35_O]^−^ (*m/z* = 303.27) and [C_16_H_36_N_6_]^−^ (*m/z* = 312.30), co-localise with cell nuclei suggesting that these ions are fragments of proteins or nucleic acids mainly present in the nucleus. [HNO_3_]^−^ (*m/z* = 62.99) and [HNO_4_]^−^ (*m/z* = 78.99) ions localise with the plasma membrane or cytosol. [C_5_H_4_N_5_]^−^ (*m/z* 134.04) is observed throughout the cell body (outside the nucleus) and also within cell-free regions of the scaffold. This ion is not present on the left-hand side of the image field which corresponds to the resin block alone, suggesting that it is associated either with collagen, the cell culture medium or molecules, such as cytokines and metabolites released by encapsulated cells. [C_4_H_8_N_3_]^−^ (*m/z* 98.07) is observed throughout the image field suggesting that this ion is not associated with the 3D culture system and originates from the epoxy resin or other components used during sample processing. The NanoSIMS images in Fig. 5c show cellular features such as the nuclear membrane and regions of highly condensed proteins within the nucleus (bright regions in the CN^−^ image) with high spatial resolution. The superior spatial resolution of NanoSIMS results, in part, from the use of high primary ion beam energies which highly fragment biomolecules limiting extraction of molecular data. However, the additional resolution is particularly powerful for localising isotopically labelled compounds with fragments derived from endogenous molecules.

**Fig. 6:**
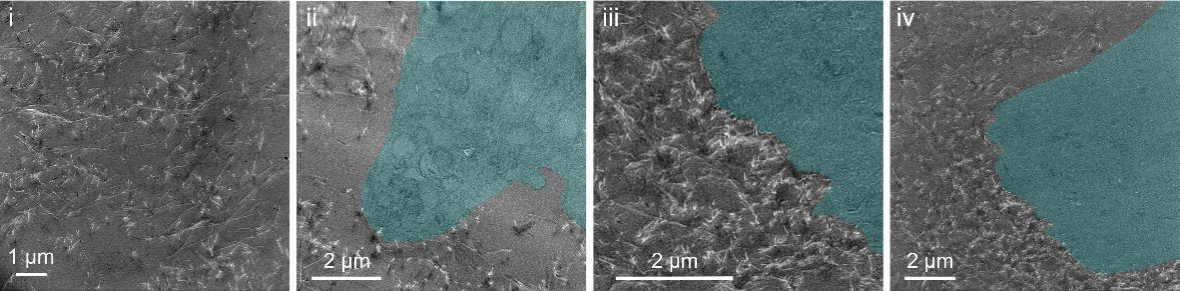
TEM micrographs of 100 nm sections of an HDF-seeded type I collagen scaffold. Cells highlighted with cyan overlays. Sections prepared from the same sample shown in optical and SIMS image data in Figs. 3a and 5.

Data from chemical and fluorescence imaging techniques enabled visualization of encapsulated of cells in the matrix, but these approaches are unable to capture the fine structure of the scaffold and cell-scaffold interfaces. To gain more insight into these features of the HDF-collagen culture system, 100 nm sections, prepared from the same samples used for optical and chemical imaging (Figs. 3a and 5), were imaged using TEM. The resulting micrographs (Fig. 6) reveal the characteristic fibrillar morphology of the collagen scaffold, with individual fibres appearing randomly oriented within a single thin (2D) section (Fig. 6i). Fibres are both sparsely (ii) and densely (iii, iv) distributed around cell protrusions. Intracellular ultrastructure, with visible compartmentalization consistent with retained cell viability, is apparent in panel ii. Since the samples used in the study were fixed after 24 h, the apparent differences in scaffold structure likely reflect differences in the spatial organisation of fibrillar collagen upon gelation. At later timepoints, degradation and replacement of the scaffold by encapsulated HDFs^47^ would be expected to result in structural changes to the scaffold detectable by TEM. Volumetric reconstruction from TEM images of serial sections would provide a means to explore these structural/morphological relationships between encapsulated cells and the scaffold in 3D.

## Discussion

We have demonstrated that an extended set of high-resolution imaging modalities can be combined to capture and analyse the physicochemical properties of cell-seeded 3D scaffolds. To our knowledge this is the first time that one and two photon excited fluorescence, SRS and SHG microscopy, ToF-SIMS, NanoSIMS and TEM have been applied to analyse a common sample. Multimodal optical microscopy image data of minimally fixed samples revealed the organisation of the actin cytoskeleton (an imaging biomarker for fibroblast activation and cell attachment to the scaffold), the relative abundance of methyl and methylene groups in endogenous molecules (proteins and lipids) within different subcellular organelles and compartments, and local variations in the density of collagen fibrils (which was observed to affect the distribution and organisation of HDFs within the scaffold). Multimodal SIMS analysis of thin resin-embedded sample sections revealed molecular fragments associated with the cell nucleus, the plasma membrane or cytosol and the scaffold and interstitial space. TEM image data showed the morphology and arrangement of individual collagen fibres and the ultrastructure of the cell-scaffold interface. Fluorescence images of the cytoskeleton were registered with label-free multiphoton images showing the distribution of CH_2_ and CH_3_ groups and collagen fibrils in the scaffold using landmarks associated with cell nuclei visible in CLSFM and TPEF images. Similarly, high mass resolution and high spatial resolution MSI data captured using ToF-SIMS and NanoSIMS systems were spatially registered using [CN]^-^ ion channel images from both platforms. In both cases the magnitude of the target registration error was found to be 250 –300 nm. Mammalian cells in 3D culture are typically 10s of µms in diameter with nuclei which are several µms in diameter. A TRE of 250 –300 nm is thus sufficient for analysis of cellular and nuclear morphology and to measure the colocalization of molecules detected in SRS and ToF-SIMS data with features, such as the cytoplasm or cell nucleus, visible in FM and NanoSIMS images. For applications, including intracellular trafficking studies, which require analysis of the co-localisation of molecules with smaller subcellular components, substantially lower TRE values would be required.

In the present study no attempt was made to spatially register correlative ToF- and NanoSIMS images with optical and TEM data. TEM and NanoSIMS have previously been applied within a correlative workflow^48^, however correlative ToF-SIMS, NanoSIMS and TEM analysis of a single section relies on optimisation of the section thickness to balance the need for adequate molecules/ions for sequential SIMS analysis with the requirement for thin sections for high-resolution TEM. With further optimisation it may be possible to acquire multichannel ToF-SIMS images from thinner sections whilst preserving the ultrastructure for TEM analysis. Analysing adjacent serial sections with SIMS and TEM would enable capture of structural and chemical information for a common cell and scaffold region. Alternatively, at the expense of reduced spatial resolution, ultrastructural information could be obtained from thicker sections using SEM prior to SIMS analysis. The topographic imprint of the grid pattern in the face of the resin block (Fig. 5a) allowed identification of the transverse ROI imaged using optical techniques. Combined with an estimate of the depth of a section within the resin block, this would permit coarse alignment of 2D SIMS, and EM image data, with the relevant slice from an optical z-stack. Landmarks based on cell features would then enable fine registration. Modifying the sample processing workflow by high pressure freezing the cell-seeded scaffold prior to freeze substitution may better preserve its ultrastructure to facilitate accurate registration of SIMS and EM data with optical images. More comprehensive registration of image data would create opportunities to apply image fusion techniques to combine image information and exploit the relative advantages of different imaging modalities, particularly in terms of spatial resolution and chemical specificity^49^.

For robustness and computational simplicity, 3D fluorescence, SRS and SHG image data was projected onto a 2D plane for spatial registration. 3D registration of these datasets may reduce target registration errors by correcting volumetric distortions, such as shearing and tilt of the sample with respect to the focal plane. As well as enabling more accurate registration, this would allow 3D co-localisation analysis between different imaging modalities. For convenience, and to facilitate accurate registration of image data captured at different times on different optical microscope systems, samples were chemically fixed prior to optical imaging. To capture dynamic behaviour, such as cell migration within the matrix, a minimally invasive optical microscopy technique, such as light sheet fluorescence microscopy^22^, could be performed prior to fixation followed by end point correlative analysis. Additionally, optical images with improved spatial resolution could be obtained using super-resolution fluorescence microscopy (SRF) techniques^23^.

We anticipate that extended correlative and multimodal imaging will have broad applications in the analysis of 3D culture systems. FM, SRS and EM are compatible with many different classes of biological samples and SIMS techniques can detect a large number of different molecular fragments derived from cell and scaffold components. Myosin, present in muscle and connective tissue, is an efficient SHG source^50^, as is the glycoprotein fibroin which is found in silk-based biomaterials^51^. There are a number of different methods for generating image contrast in biomaterials which lack the non-zero second order susceptibility required for SHG, including Raman scattering^52^, autofluorescence^53^ and the addition of exogenous fluorescent tags applied to stain scaffold fibres before^54^ or after assembly^55^. SRS and SIMS are particularly powerful for visualizing and quantifying the distribution and intracellular uptake of compounds containing specific chemical bonds or molecular fragments which enable them to be distinguished from endogenous species^56^. NanoSIMS can be used to track the fate of the isotopically labeled compounds in pulse-chase experiments through acquisition of pairs of stable isotope images and derivation of the isotope ratio image. The distribution of the isotopically-labeled compound is then determined from those image regions where the isotope ratio is in exceeds its natural value^57^. Combining this spatially-resolved chemical information with the biomolecular specificity of FM and the ultrastructural information accessible to TEM, such integrated, correlative analysis offers a way to analyse the effects of therapeutics and cytotoxic compounds^58^ over subcellular to multicellular length scales.

## Methods

### Cell culture and hydrogel preparation

**Fig. 7:**
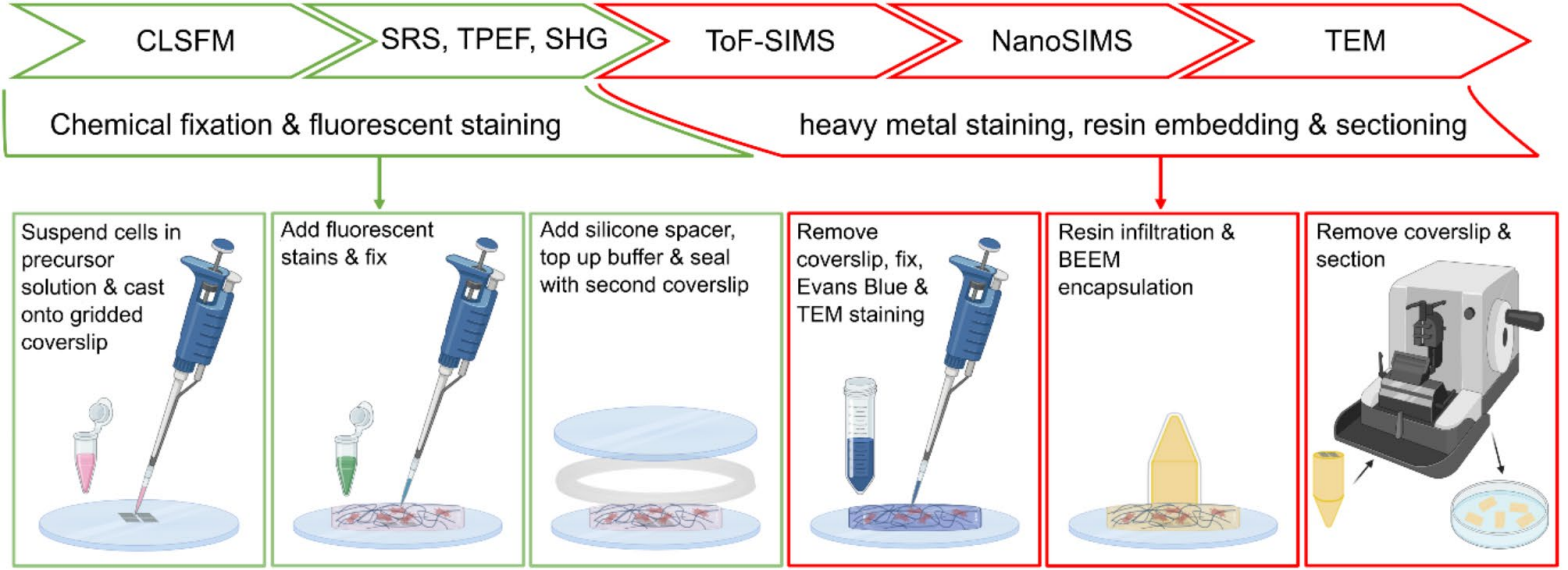
Overview of sample preparation steps. Collagen gels seeded with HDFs were cast on gridded coverslips and stained for fluorescence microscopy. A silicone spacer ring was added before the sample was encapsulated under a second coverslip. For SIMS and TEM analysis additional contrast agents were added before resin embedding and sectioning.

Figure 7 presents an overview of the steps used to prepare type I collagen scaffolds seeded with HDFs for correlative imaging. HDFs (Merck, UK) were maintained in 1x Dulbecco’s Modified Eagle’s Medium (DMEM) supplemented with 10% v/v fetal bovine serum (FBS) and antibiotics (gentamicin and amphotericin B) at 37 °C, 5% CO2 and 95% humidity. Cells above 80% confluency were washed three times with phosphate buffered saline (PBS) and trypsinized, before addition of serum supplemented media to eliminate the secondary toxic effects of trypsin. Detached cells were spun down by centrifugation, and the excess solvent was replaced by cell growth media. Cells were counted before seeding in gels. Collagen type I from rat tail (5 mg/ml IBIDI, UK) hydrogel was prepared according to manufacturer instructions at 1.5 mg/ml concentration. Cells were mixed in gel precursor solution at a density of 500 cells per µl of gel and a 300 µl gel was cast thinly on a gridded glass coverslip (Grid-50, IBIDI, UK). After 24-hours, the HDFs encapsulated in the gel were stained for F-actin (CellMask Actin Tracking Deep Red, Thermofisher) and nuclei (Hoechst 33342, Thermofisher). The actin stain was diluted 1:1000 in Opti-MEM (Gibco, Thermofisher) and the nuclear stain was diluted to 0.01 mg/ml in Opti-MEM before addition to the cell-seeded hydrogel. After 1 h at 37⁰C, 5% CO_2_, the sample was washed 3 times with 1x PBS and fixed for 30 min at room temperature using 4% paraformaldehyde solution (Thermofisher, UK). The fixative was then washed off by rinsing the gel 3 times with 1x PBS.

To mount gels for optical (CLSF, TPEF, SRS and SHG) microscopy (see Fig. S3 in supplementary material), an annulus with an outer diameter of approximately 28 mm was cut from a 125 μm thick silicone sheet (NA500-1 type, Nagor) and placed on the coverslip around the fixed gel. The gel was hydrated with 1x PBS and a second coverslip was placed on top to ensure that the gel remained sealed and hydrated during imaging. Following acquisition of optical microscopy image data, resin-embedded sample blocks were prepared for SIMS and TEM analysis. The top coverslip and silicone ring were gently removed, and the sample was prepared for resin embedding following a previously published protocol^59^. Briefly, the sample was post-fixed using 1% glutaraldehyde solution before staining with a 2 mg/ml solution of Evans Blue (Sigma Aldrich) for an hour at room temperature to identify the clear hydrogel sample within the larger resin block. Further staining was performed with 2% (v/v) osmium tetroxide (Agar Scientific) in 0.2 M cacodylate buffer (Agar Scientific) containing 1.5% (w/v) potassium ferricyanide (Acros Organics). The sample was then incubated in 2% (w/v) uranyl acetate (Agar Scientific) and dehydrated using a graded ethanol series (Fisher Scientific). Epoxy Durcupan ACM resin (Sigma Aldrich) was prepared and mixed with acetone for sample infiltration. After overnight infiltration of resin, a BEEM conical tip capsule (Agar Scientific) was filled with ACM resin and the capsule was placed on top of the hydrogel coverslip before polymerization at 60 °C for 48 h. The coverslip was removed from the hardened resin by plunging into liquid nitrogen leaving a relief print of the grid squares and identifiers on the resin surface. For SIMS analysis, 250 nm sections were cut from the resin blocks using an ultramicrotome (Leica EM UC7) with a Histo Jumbo diamond knife (Agar Scientific) moving at a speed of 1 mm/s. Sections were collected on cleaned 5 × 5 mm doped silicon chips and dried on a hot plate. For TEM, 100 nm sections were cut similarly and collected onto TEM grids.

### Confocal laser scanning fluorescence microscopy

Fluorescence microscopy images were acquired using a confocal laser scanning microscope (Stellaris 5, Leica Microsystems) with a 63x / 1.4 NA oil immersion objective lens. Cell nuclei and actin filaments were imaged under excitation at 405 nm and 647 nm with an emission bandpass of 420 –533 nm and 658 –776 nm respectively. Laser power and detector gain were set to optimize the quality of images over the first ~ 200 μm of the gel. In practice this meant partially saturating the detector signal when imaging close to the gel-coverslip interface in order to obtain a suitable signal-to-noise ratio for images captured deeper into the gel, where the emitted fluorescence was weaker due to light absorption, scattering and the spherical aberration introduced by the refractive index mismatch between the immersion oil (*n* = 1.52) and the hydrogel (*n* ~ 1.38). An extended volume of the sample was imaged by stitching together a series of overlapping focal series (z-stacks) using the microscope control software (LAS-X, version 4.7.0.28176). After stitching, the intensity of each image slice was scaled to correct the reduction in brightness with depth using an exponential decay curve fitted to the entire image stack.

### Two photon excited fluorescence, stimulated Raman scattering and second harmonic generation microscopy

SRS, SHG and TPEF microscopy images were acquired using a laser scanning microscope (SP8, Leica Microsystems) coupled to a PicoEmerald-S laser system (APE). The PicoEmerald-S generates two pulsed 2 ps laser beams: a 1031.2 nm Stokes beam which was spatially and temporally overlapped with a pump beam which was tuned from 795 to 810 nm. Methylene and methyl groups were imaged using SRS with the pump beam set to 798 nm (corresponding to the 2850 cm^− 1^ symmetric CH_2_ stretch) and 791 nm (corresponding to the 2945 cm^− 1^ asymmetric CH_3_ stretch). The Stokes beam was modulated at 20 MHz and stimulated Raman loss signals were detected using a silicon-based detector and lock-in amplifier (UHFLI, Zurich instruments). SHG and TPEF signals were recorded under excitation at 1031 nm using dedicated emission filters (LL01-514-25 from Semrock and BP670/125 from Chroma). Images were acquired with a 25x/1.1 water immersion objective and a short working distance air condenser lens (0.9 NA). The laser power was set to 70% which corresponded to approximately 26 mW for the pump beam and 68 mW for the Stokes beam at the sample. Detector gain settings were consistently applied across the study (7.8 V for SRS, 1277.9 V for SHG and 588.8 V for TPEF). Large area mosaic tile scanning was performed using LAS-X ‘Navigator’ during which 512 × 512 pixel images were acquired for each tile at X1 zoom with an imaging speed of 400 Hz in order to identify the same region of interest (ROI) imaged using CLSFM. Following acquisition, images were processed offline using ImageJ^60^ (version 1.54 h). Off-resonance (spurious) SRS signal contributions were subtracted from their on-resonance counterparts in a pixel-by-pixel manner using the ‘Image calculator’ plugin.

### Secondary ion mass spectrometry imaging

ToF-SIMS images were acquired using a ToF-SIMS 5 system (ION-TOF GmbH) equipped with a time-of-flight mass (ToF) mass analyzer. All data were acquired using the ToF analyzer and a 30 keV Bi_3_^+^ Liquid Metal Ion Gun (LMIG) as a primary ion source. 75 μm × 75 μm (512 × 512, 146.5 nm pixels) images were captured in negative polarity using random rastering with an ion current of 0.03 pA. An electron floodgun (with an electron current of -7 µA) was used to compensate charging over the surface of the sample. Acquisition was stopped after 55 scans, corresponding to a primary ion dose of 3.84 × 10^13^ ions/cm^2^. Data were acquired and analyzed using SurfaceLab 7 (version 7.3.137385) and converted into BiFF 6 format for further analysis. The mass scale was internally calibrated using signals from [C]^−^, [CH]^−^, [CH_2_]^−^, [C_2_]^−^, and [C_2_H]^−^ ions, identified by peak position, isotopic distribution of recognisable species, and the structure of the mass spectrum. Putative assignment of subsequent species was based on the closest match of the peak to the theoretical mass of ions or molecular fragments expected to be present in the sample.

Following ToF-SIMS data acquisition, the same ROI was analysed with high spatial resolution using a Cameca NanoSIMS 50 L system (Cameca). Reflected light images from an integrated optical microscope were used to identify the sputter crater left by the ToF-SIMS instrument, before the sample position was fine-tuned using an ion induced secondary electron image in order to acquire isotopic SIMS data. 35 × 35 μm (512 × 512, 68.4 nm pixels) images were captured using a 16 keV impact energy primary beam of Cs^+^ ions. Negative secondary ions for^16^O, ^12^C_2_, ^12^C^14^N, ^31^P and^32^S were measured with a primary beam dwell time of 5 ms/pixel. The D1 aperture was set at position 4 (150 μm diameter) and an L1 voltage of 2000 V was applied to boost the primary beam current impinging on the sample to 0.54 pA. A total of 5 image planes were collected resulting in an image acquisition time of 110 min. No entrance or aperture slit was used resulting in low mass resolving power. In cases where entrance and exit slits are required to achieve high mass resolution, the acquisition time can simply be lengthened, or a larger D1 aperture used to increase the primary beam current and secondary ion acceptance diameter into the mass spectrometer, albeit at the expense of image spatial resolution. Acquired image data were processed to correct drift between sequential image frames before summing^57^ using ImageJ^60^ (version 1.51j) and the OpenMIMS plugin (version 2.8.0). Peaks in the mass spectrum were identified based on isotope ratios (the accuracy of which is typically a few ‰) and by mass differences between known peaks and adjacent peaks at expected nominal masses^61^.

### Transmission electron microscopy

100 nm sections of resin embedded cell-seeded scaffolds were imaged using a Talos Arctica (FEI Thermo Fisher Scientific) TEM. 4096 × 4096 pixel images were captured using a Ceta 16 M camera with an integration time of 2 s with an accelerating voltage of 200 kV. At magnifications of between 3,400x and 5,300, the FoVs varied between 18 μm x 18 μm and 11 μm x 11 μm.

### Image registration

Maximum intensity (z) projections were computed from optical microscopy image stacks cropped to the same axial range within the sample. After upsampling SRS, TPEF and SHG images by a factor of 5.1 to match the CLSFM pixel size, image channels in which the coverslip grid was visible were analysed to estimate a rigid body transform to coarsely align the datasets. Fine registration was then performed using cell nuclei, visible in TPEF and CLSFM images, as fiducials. The scale invariant feature transform (SIFT)^62^, implemented in the imageJ plugin “Extract SIFT correspondences” (version 2.0.3) was used to identify common keypoints in both images. After candidates were filtered using RANSAC, with a predefined maximal alignment error of 25 pixels, the remaining (typically 5–10) keypoints were used to register the TPEF image to the corresponding CLSFM image channels using ImageJ’s “bUnwarpJ” plugin^63^ (version 2.6.13). The same transform was subsequently applied to the SRS and SHG image channels before a further rigid body transform was applied to minimize the mean square error^64^ and correct small chromatic offsets between the CH_2_, CH_3_ and TPEF images. Finally, a scaled copy of the TPEF-Hoechst image channel was subtracted from the SHG channel to facilitate visualization of the SHG-collagen signal close to cell nuclei.

A similar method was used to register NanoSIMS and ToF-SIMS images of resin embedded sections. Common keypoints were identified in the^12^C^14^N channel of the NanoSIMS data and the ToF-SIMS channel corresponding to cyanide ([CN]^−^) ions, a mass to charge ratio (*m/z*) of 26. This ion is abundant throughout the cell body and, in particular, within the nuclear membrane. ToF-SIMS images were upsampled by a factor of 2.1 to match the pixel size of the NanoSIMS images before manual cropping and rotation to coarsely align the images. Keypoints were identified using SIFT to derive an elastic transform to register the ToF-SIMS image data to the NanoSIMS images. For illustration, Fig. S4 (see supplementary material) shows the seven keypoints used to register the optical images in Fig. 3a and the eight keypoints used to register the SIMS images in Fig. 5.

## Electronic supplementary material

Below is the link to the electronic supplementary material.

Supplementary Material 1.

## Data Availability

The image data presented in this article are available in the figshare repository https://figshare.com/s/c0007f8e30c094e47dee .
