# Supplementary material for: Multiparametric physicochemical analysis of a type 1 collagen 3D cell culture model using light and electron microscopy and mass spectrometry imaging
Source: Sci Rep. 2025 Mar 20;15:9578. doi: 10.1038/s41598-025-93700-3 (PMC11926111; doi:10.1038/s41598-025-93700-3)
Supplement: Supplementary file 1 — Supplementary Material 1. [file 41598_2025_93700_MOESM1_ESM.pdf]

Multiparametric physicochemical analysis of a type 1 collagen 3D cell culture model using light and electron microscopy and mass spectrometry imaging: supplementary information

Camilla Dondi[1], Dimitrios Tsikritsis[1], Jean-Luc Vorng[1], Gina Greenidge[1], Ibolya Kepiro[1], Natalie A. Belsey[1,2], Greg McMahon[1], Ian S. Gilmore[1], Maxim G. Ryadnov[1], & Michael Shaw[1,3]*

[1]National Physical Laboratory, Hampton Road, Teddington, TW11 0LW, UK
[2]School of Chemistry & Chemical Engineering, University of Surrey, Guildford GU2 7XH, UK
[3]Department of Computer Science, University College London, Gower Street, London, WC1E 6BT, UK
*mike.shaw@npl.co.uk

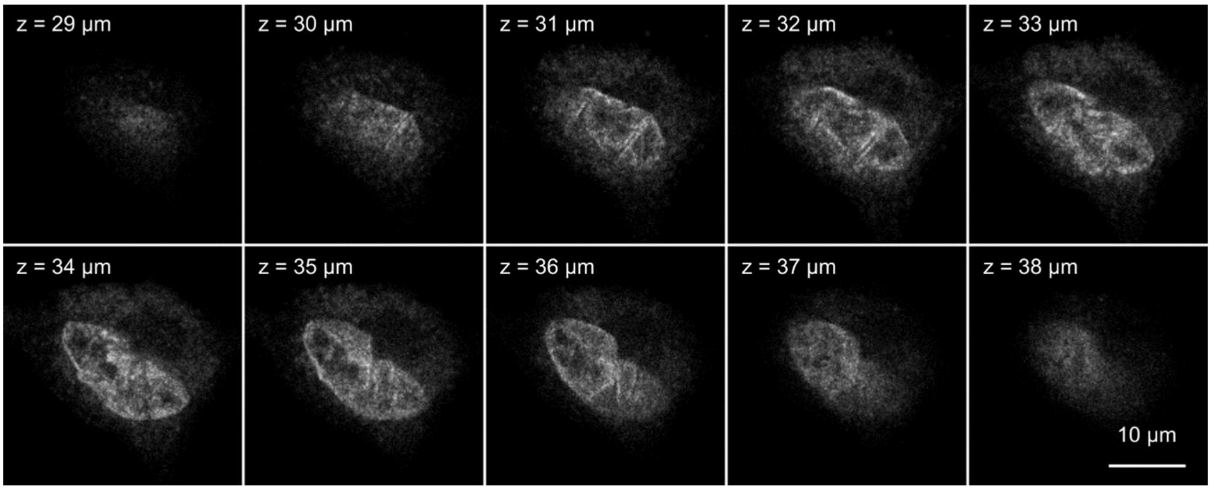

Figure S1. Sequential slices from a CLSFM z-stack showing the nucleus of the cell highlighted in Fig. 4(b) of the main manuscript.

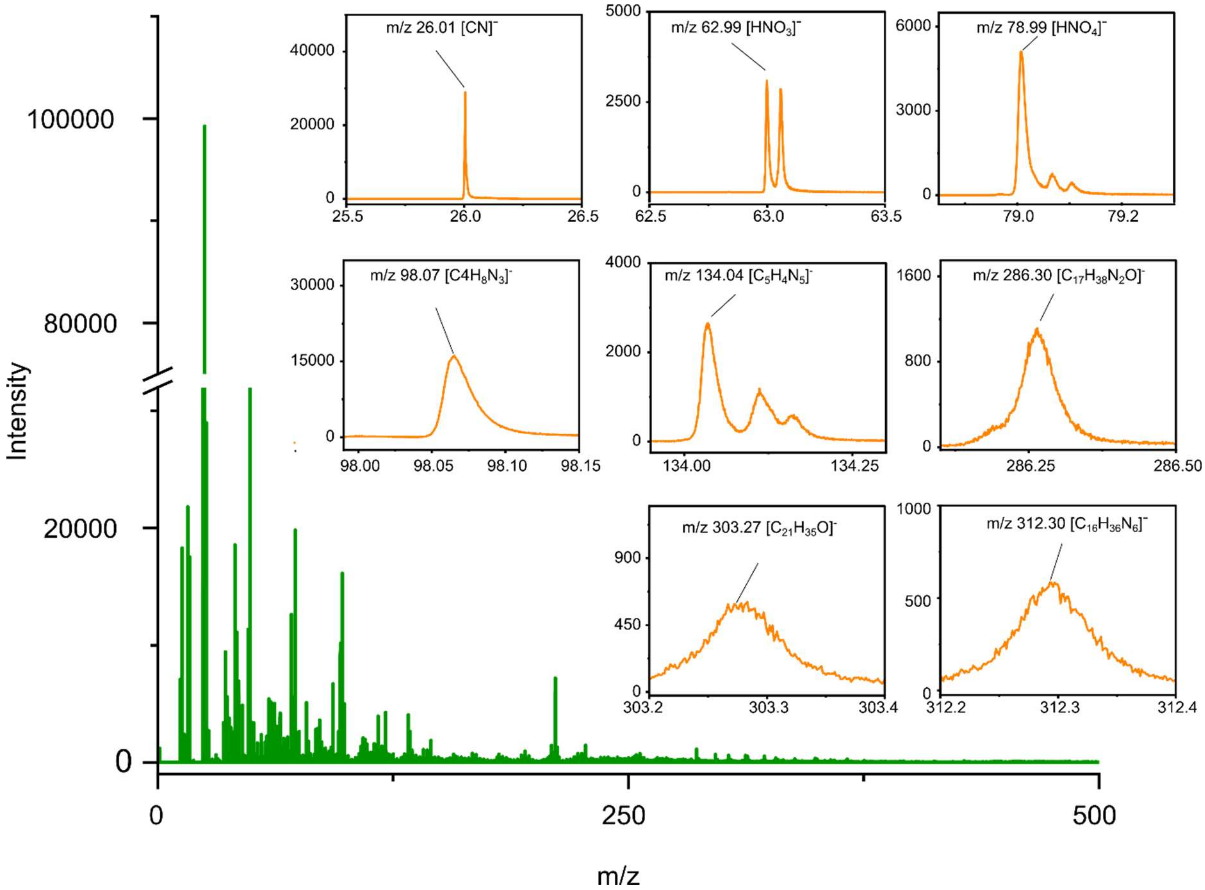

Figure S2. ToF-SIMS mass spectrum corresponding to the image field shown in Fig. 5(b) of the manuscript. *Insets show the spectra corresponding to the individual ion images shown the figure.*

**Experimental mass**	**Theoretical mass**	**Assignment**	**Mass deviation (ppm)**
26.01	26.00	$[CN]^-$	76.91
62.99	63.00	$[HNO_3]^-$	-79.37
78.99	78.99	$[HNO_4]^-$	12.65
98.07	98.07	$[C_4H_8N_3]^-$	-20.39
134.04	134.05	$[C_5H_4N_5]^-$	-52.22
286.30	286.30	$[C_{17}H_{38}N_2O]^-$	6.98
303.27	303.27	$[C_{21}H_{35}O]^-$	3.29
312.30	312.30	$[C_{16}H_{36}N_6]^-$	-3.20

Table S1. Measured peak positions, theoretical masses of the assigned ions and mass deviation corresponding to the ToF-SIMS image channels shown in Fig. 5(b) of the manuscript.

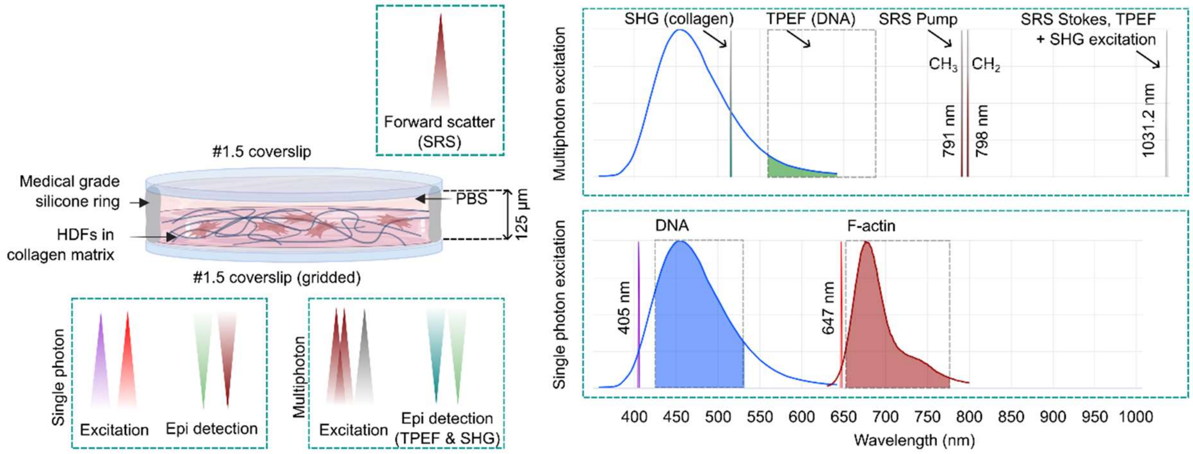

Figure S3. Sample mounting and imaging geometry for optical microscopy techniques used in the correlative imaging workflow. *Type I collagen scaffolds seeded with HDFs were mounted between two #1.5 coverslips separated by a silicone spacer ring to allow illumination and light collection using oil immersion objective and condenser lenses for SRS imaging and reduce evaporation and maintain sample structure during multimodal optical imaging.*

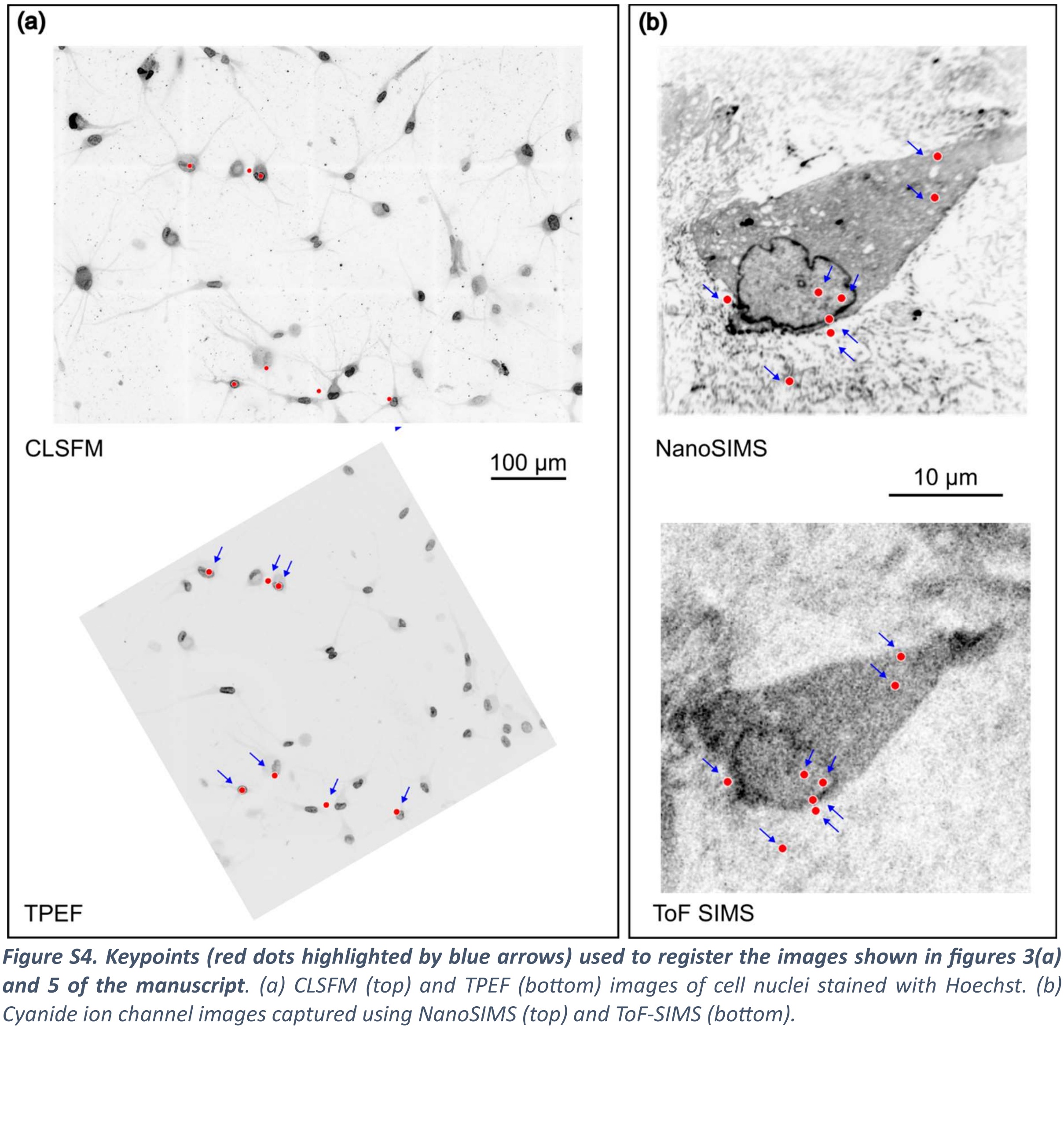

Figure S4. Keypoints (red dots highlighted by blue arrows) used to register the images shown in figures 3(a) and 5 of the manuscript*. (a) CLSFM (top) and TPEF (bottom) images of cell nuclei stained with Hoechst. (b) Cyanide ion channel images captured using NanoSIMS (top) and ToF-SIMS (bottom).*
